# Identification and Analysis of Differentially-Expressed microRNAs in Japanese Encephalitis Virus-Infected PK-15 Cells with Deep Sequencing

**DOI:** 10.3390/ijms16012204

**Published:** 2015-01-20

**Authors:** Yuhan Cai, Ling Zhu, Yuanchen Zhou, Xiao Liu, Xiaowan Liu, Xinqiong Li, Qiaoli Lang, Xiaogai Qiao, Zhiwen Xu

**Affiliations:** 1 Animal Biotechnology Center, College of Veterinary Medicine, Sichuan Agricultural University, Ya’an 625014, China; E-Mails: caiyuhan429197524@126.com (Y.C.); abtczl72@126.com (L.Z.); abtczyc@163.com (Y.Z.); abtclhl@126.com (X.L.); abtclxw@126.com (X.L.); abtclxq@126.com(X.L.); langqiaoli77@126.com (Q.L.); abtcwzfb@126.com (X.Q.); 2 Key Laboratory of Animal Disease and Human Health, College of Veterinary Medicine, Sichuan Agricultural University, Ya’an 625014, China

**Keywords:** Japanese encephalitis virus, microRNA, pathogenesis, deep sequencing

## Abstract

Japanese encephalitis virus (JEV), a mosquito-borne *Flavivirus*, causes acute viral encephalitis with high morbidity and mortality in humans and animals. MicroRNAs (miRNAs) are small noncoding RNAs that are important modulators of the intricate host-pathogen interaction networks. However, our knowledge of the changes that occur in miRNAs in host cells after JEV infection is still limited. To understand the molecular pathogenesis of JEV at the level of posttranscriptional regulation, we used Illumina deep sequencing to sequence two small RNA libraries prepared from PK-15 cells before and after JEV infection. We identified 522 and 427 miRNAs in the infected and uninfected cells, respectively. Overall, 132 miRNAs were expressed significantly differently after challenge with JEV: 78 were upregulated and 54 downregulated. The sequencing results for selected miRNAs were confirmed with RT-qPCR. GO analysis of the host target genes revealed that these dysregulated miRNAs are involved in complex cellular pathways, including the metabolic pathway, inflammatory response and immune response. To our knowledge, this is the first report of the comparative expression of miRNAs in PK-15 cells after JEV infection. Our findings will underpin further studies of miRNAs’ roles in JEV replication and identify potential candidates for antiviral therapies against JEV.

## 1. Introduction

Japanese encephalitis virus (JEV), a mosquito-borne member of the genus *Flavivirus*, is the most important causative agent of acute viral encephalitis in humans, occurring mainly in Asian countries [1]. Approximately 50,000 cases of JEV occur per year worldwide, resulting in more than 10,000 deaths and 15,000 cases of neurological or psychiatric sequelae [2]. However, the actual number of JEV infections is probably much higher, possibly up to 175,000 annually [3]. Pigs act as amplifying hosts of JEV; therefore, the domestic pig was considered to be a risk factor in the transmission of the disease to humans. JEV is also one of the main causes of infectious reproductive failure in swine, resulting in significant economic losses in the swine industry [1,4]. To effectively control JEV infection, it is essential to clarify the mechanisms of virus pathogenesis.

MicroRNAs (miRNAs), which can be produced by both hosts and viruses, are an abundant class of short (~22 nt), endogenously-encoded, single-stranded RNAs involved in the posttranscriptional regulation of gene expression [5]. They can induce mRNA degradation or translational repression by binding to perfect or near-perfect complementary target sequences, usually located in the 3' untranslated regions (3'UTRs) of transcripts [6,7,8]. miRNAs regulate gene expression in a wide range of biological processes, including developmental timing, signal transduction, tumorigenesis, innate and adaptive immunity, cell proliferation and apoptosis [9,10,11]. Recent studies have focused on the roles of miRNAs as modulators of host cell-virus interaction networks [12,13]. Because the propagation of a virus is highly dependent on its host cell, the modification of the complex cellular regulatory networks by cellular miRNAs can greatly influence viral reproduction and pathogenesis. In recent years, increasing numbers of studies have reported the impact of viral infections on the cellular miRNAome; for example, the expression levels of 108 human miRNAs were shown to change more than 2.0-fold in hepatitis C virus (HCV)-infected human hepatoma cells, and the differentially-expressed miRNAs, including miR-24, miR-149*, miR-638 and miR-1181, were shown to be involved in virus entry, replication and propagation [14]. Infection of porcine dendritic cells with the alphaherpesvirus pseudorabies virus alters several cellular miRNAs, including miR-27b*, miR-29a and miR-30e-3p. *In silico* target analysis of these differentially-expressed miRNAs suggests that they may be involved in viral latency [15].

Although little is yet known about the involvement of miRNAs in JEV infection, three recent studies suggested a role for miRNA-mediated gene regulation in the pathogenesis of JEV. Pareek* et al.* [16] reported that miR-155 is upregulated in JEV-infected human microglial cells and that its overexpression enhances CD45 expression, reduces the expression of complement factor H (CFH) and proinflammatory cytokines and suppresses JEV replication. However, Thounaojam* et al.* [17] demonstrated the opposite role for miR-155 in modulating JEV replication, suggesting that miR-155 plays a proinflammatory role in JEV-infected BV-2 cells (a mouse microglia cell line), primary microglial cells and in both the mouse and human brains. Although a major difference between these two studies was the investigation of different kinds of cells and viral strains, the contradictory results may also be attributable to the varying increases in miR-155 during JEV infection. miR-29b is reported to regulate JEV-induced microglial activation by inhibiting the expression of the anti-inflammatory protein, TNFAIP3, which results in the sustained activation of NF-κB, in JEV-infected BV-2 cells and primary microglial cells [18]. 

However, no comprehensive data on the differential expression of miRNAs in the host during JEV infection have yet been reported. The aim of this study was to characterize the miRNA regulation in PK-15 cells during the course of JEV infection using an Illumina/Solexa deep sequencing approach, to increase our understanding of the pathogenesis of the virus and to identify new diagnostic and therapeutic approaches to viral diseases.

## 2. Results and Discussion

### 2.1. Overview of the Deep Sequencing Results

**Figure 1:**
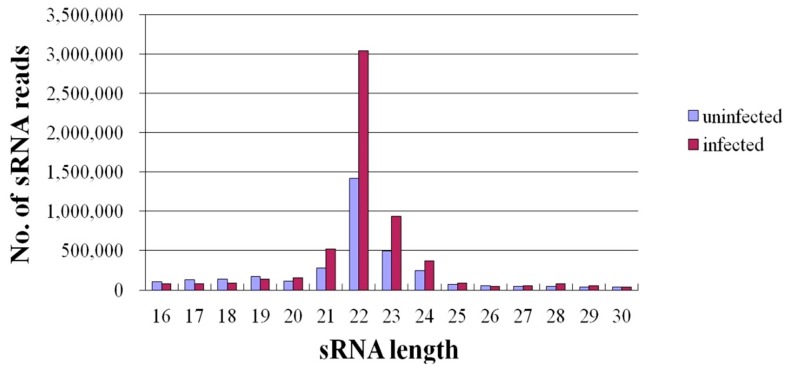
Length distributions of small RNAs in Japanese encephalitis virus (JEV)-infected and -uninfected PK-15 cells.

In recent years, deep sequencing has become a powerful strategy for identifying novel miRNAs that cannot to be identified with microarrays and studying the expression profiles of miRNA in different species at various developmental stages, in normal and diseased states, *etc*. [19,20]. After JEV follows its natural route of infection via JEV-carrying mosquitoes, the virus first multiplies in skin epithelial cells and lymph nodes, infects peripheral organs, such as kidney, liver and spleen, and then invades the subsequent transient viremia, after which the neurotropic virus spreads to the CNS [21]. PK-15 cells, a porcine kidney epithelial cell line, have a similar function and susceptibility as skin epithelial cells. In addition, numerous studies on JEV have been conducted in PK-15 cells [22,23,24]. Therefore, PK-15 cells are a good model in which to evaluate the role of cellular miRNAs in the host response to JEV infection. To investigate the effects of JEV infection on miRNA expression, two small-RNA libraries from infected and uninfected PK-15 cells were constructed and sequenced with deep sequencing (Solexa/Illumina) technology. In total, 6,290,138 and 3,987,148 filtered high-quality reads were obtained from the infected and uninfected libraries, respectively. Reads with copy numbers of ≤2 and that were shorter than 15 bases after the adapters were trimmed were deleted. The total numbers of clean reads obtained from each library were then 5,926,967 and 3,562,868, respectively. The size distributions of the small RNAs were similar in the infected and uninfected libraries. Most of the small RNAs (67.86%–82.11%) from both libraries were 21–24 nt in length, and 22-nt RNAs were the most abundant, accounting for 51.27% and 39.69% of the small RNAs in the infected and uninfected libraries, respectively (Figure 1). This is consistent with the typical size of miRNA from Dicer-derived products, and our results suggest that the libraries were highly enriched in miRNA sequences. We also excluded certain known types of RNA sequences, including mRNA, rRNA, tRNA, snRNA, snoRNA and repetitive sequence elements. Ultimately, 5,106,674 and 2,876,659 clean reads remained from the infected and uninfected libraries, respectively, and were used for the miRNA analysis.

### 2.2. Expression of miRNAs in JEV-Infected and -Uninfected PK-15 Cells

The miRNAs frequently varied from their miRBase reference sequences, displaying multiple mature isoforms (designated “isomiRs”). When we compared all of the isomiRs in our libraries, 3' heterogeneity was more common than 5' heterogeneity. This is consistent with the findings of other miRNA deep sequencing studies and the observation that most variability occurs in either the Dicer1 or Drosha cleavage position within the pre-miRNA hairpin [15,25]. To ensure the most robust comparison of miRNA expression across libraries, the most abundant isomiR, rather than the miRBase reference sequence, was chosen as the reference sequence in this study [26].

**Figure 2:**
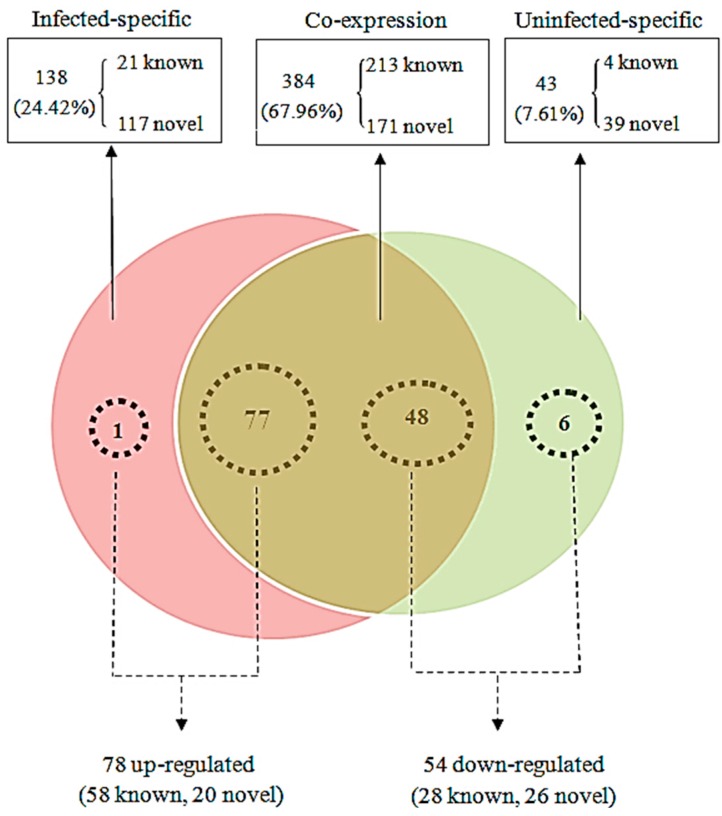
Comparison of differentially-expressed miRNAs between the JEV-infected and -uninfected cells. The Venn diagram displays the distribution of 565 unique miRNAs across the infected group and uninfected group. The dashed circles indicate the miRNAs that were significantly differentially expressed in the infected group relative to the uninfected group.

After mapping the clean reads to the relevant bioinformatics software, as described in the Materials and Methods, 565 miRNAs were identified in this study. Among these, 384 miRNAs were found in both libraries, whereas 138 miRNAs were found only in the JEV-infected group and 43 miRNAs were unique to the JEV-uninfected group (Figure 2, Table S1 in Supplementary Material). The majority of both known and novel miRNAs had lengths of 22, 21 or 23 nt. Analysis of the first nucleotide bias in the 238 known and 327 novel miRNAs is presented in Figure S1 in Supplementary Material. The miRNAs showed different biases. Uridine (U) dominated the first position, whereas guanine (G) was the least favored first base, in both the known and novel miRNAs in this study, which is a characteristic feature of miRNAs.

In both libraries, most miRNAs with abundant counts were represented by only a few miRNAs. The 10 most strongly-expressed miRNAs are listed in Table 1 and accounted for 80.31% and 84.91% of the total most abundant miRNA reads in the infected and uninfected cells, respectively. Among the 10 most abundant miRNAs, six were present in both libraries. The most strongly expressed miRNA in both libraries was miR-21, which represented 57.63% and 62.25% of the total most abundant miRNA reads in the infected and uninfected groups, respectively. This is consistent with previous reports that miR-21 is ubiquitously expressed in various cell types under normal conditions and is expressed at high levels during viral infection and that it may be related to the cell cycle in cell differentiation pathways [15,27,28]. During miRNA maturation, one arm within the precursor sequence is preferentially selected for inclusion in the mature miRNA [29]. The suffixes 5p and 3p denote miRNAs that are derived from the 5' and 3' arms of the same pre-miRNAs, respectively. In both libraries, the frequency of miRNAs with the suffix 5p was generally higher than the frequency of those with the suffix 3p (Table S1).

**Table 1:** Ten miRNAs most strongly expressed in JEV-infected and -uninfected PK-15 cells.

Ranking	JEV-Infected Group		JEV-Uninfected Group	
	miRNA Reads		miRNA Reads	
1	ssc-miR-21	17,39,040	ssc-miR-21	877,629
2	ssc-let-7f	309,868	ssc-let-7f	151,697
3	ssc-miR-30a-5p	69,597	ssc-miR-19b	33,441
4	ssc-miR-100	60,186	ssc-miR-24-3p	23,501
5	ssc-miR-29a	53,334	ssc-miR-152	22,650
6	ssc-miR-152	49,317	ssc-miR-18a	21,872
7	ssc-miR-10a-5p	39,632	ssc-let-7a	20,908
8	ssc-miR-19b	37,389	ssc-miR-100	16,274
9	ssc-miR-26a	35,650	ssc-miR-19a	14,533
10	ssc-miR-182	29,255	ssc-miR-30a-5p	14,489

### 2.3. Differentially-Expressed miRNAs in JEV-Infected PK-15 Cells

**Figure 3:**
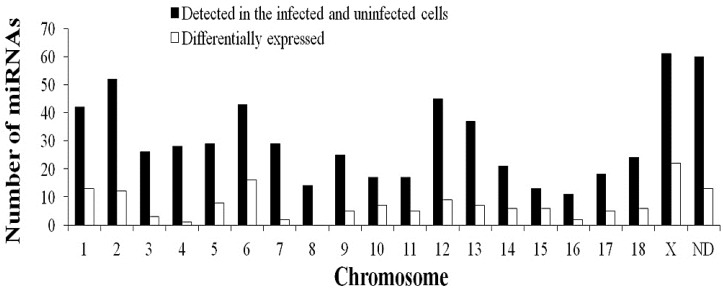
Chromosomal locations of miRNAs based on the numbers of total miRNAs (detected in the infected and uninfected cells) and differentially-expressed miRNAs. “ND” means that the genome location of the pre-miRNA has not been determined.

When a host is infected with a viral pathogen, it produces a strong antiviral response to protect itself. To combat this response, the virus induces a response that facilitates its own replication. The precise changes in the microenvironment inside the host cell caused by viral infection require the exquisite regulation of cellular miRNA expression. In total, 132 miRNAs (including 86 known miRNAs and 46 novel miRNAs) were identified as differentially expressed by ≥2-fold. Of these 132 dysregulated miRNAs, 78 miRNAs (one infection-specific, 77 co-expressed) were upregulated and 54 (six infection-specific, 48 co-expressed) were downregulated in the infected cells relative to the uninfected cells (Figure 2, Table S2 in Supplementary Material). The chromosomal distributions of the 132 unique dysregulated miRNAs corresponding to 148 dysregulated pre-miRNAs are shown in Figure 3. These 148 pre-miRNAs were distributed across nearly all of the pig (*Sus scrofa*) chromosomes (SSC1–SSC18 and SSCX), excluding SSC8. miRNA expression, determined as the number of dysregulated pre-miRNAs per chromosome, was greatest from SSCX. Of 61 miRNAs located on SSCX that were detected in the infected and uninfected cells, 22 (36.07%) miRNAs have been defined as dysregulated miRNAs. Then, 16 (of 43, 37.21%) dysregulated were located on SSC6.

When a host is infected with a viral pathogen, it produces a strong antiviral response to protect itself. To combat this response, the virus induces a response that facilitates its own replication. The precise changes in the microenvironment inside the host cell caused by viral infection require the exquisite regulation of cellular miRNA expression. In total, 132 miRNAs (including 86 known miRNAs and 46 novel miRNAs) were identified as differentially expressed by ≥2-fold. Of these 132 dysregulated miRNAs, 78 miRNAs (one infection-specific, 77 co-expressed) were upregulated and 54 (six infection-specific, 48 co-expressed) were downregulated in the infected cells relative to the uninfected cells (Figure 2, Table S2 in Supplementary Material). The chromosomal distributions of the 132 unique dysregulated miRNAs corresponding to 148 dysregulated pre-miRNAs are shown in Figure 3. These 148 pre-miRNAs were distributed across nearly all of the pig (*Sus scrofa*) chromosomes (SSC1–SSC18 and SSCX), excluding SSC8. miRNA expression, determined as the number of dysregulated pre-miRNAs per chromosome, was greatest from SSCX. Of 61 miRNAs located on SSCX that were detected in the infected and uninfected cells, 22 (36.07%) miRNAs have been defined as dysregulated miRNAs. Then, 16 (of 43, 37.21%) dysregulated were located on SSC6.

Of the 132 dysregulated miRNAs in this study, several have already been reported in association with cellular responses to viral infection. For instance, PRRSV downregulates the expression of miR-125b post-infected MARC-145 cells and activates NF-κB to facilitate its own multiplication [30]; while the downregulation of miR-24 in PRRSV-infected PAMs (Primary alveolar macrophages) increased the expression of IRG6 and the subsequent induction of the antiviral response [27]. The upregulation of miR-155 in murine peritoneal macrophages after challenge with vesicular stomatitis virus (VSV) feedback positively regulates the host antiviral innate immune response by promoting type I interferon (IFN) signaling by targeting *SOCS1*, thus suppressing viral replication [31]. miR-122 was strongly upregulated in HCV-associated hepatocellular carcinoma, suggesting that it downregulates the target mRNA of a yet-to-be-determined tumor suppressor gene, enhancing tumor growth [32]. Another study demonstrated that miR-122 has anti-inflammatory and antitumorigenic functions [33]. Moreover, the upregulation of miR-142-5p, miR-221, miR-30, miR-32, miR-374, miR-99a, miR-122 and miR-101 and the downregulation of miR-145, miR-195 and miR-98 observed in JEV-infected PK-15 cells in our study have been reported in the brains of mice infected with West Nile virus (WNV), another mosquito-borne flavivirus [34]. These findings suggest that the modulation of cellular miRNA expression during JEV infection influences viral replication, which ultimately determines the fate of the host and the disease outcome.

The miRNA miR-18a was the most strongly regulated by JEV infection in PK-15 cells. Dicer is a proven target of miR-18a [35]. Dicer modulates miRNA biogenesis and, thus, controls the output of mature miRNAs. Deficiencies in Dicer lead to increased virus production, such as is observed in HIV and VSV infections, attributable to the suppression of certain host miRNAs with antiviral activity [36,37]. In addition, Dicer plays an important role in immune cell development [38,39]. Therefore, one of the possible functions of miR-18a downregulation during JEV infections may be to increase the expression of Dicer and subsequently inhibit JEV replication. In addition to Dicer, TNFAIP3 and PIAS3 are other proven targets of miR-18a. With the reduction in miR-18a, the mRNA levels of TNFAIP3 and PIAS3 were upregulated, dampening the inflammatory response, which may contribute to better viral clearance and increased protection against JEV [40,41,42,43]. These results suggest that the downregulation of miR-18a may be associated with the strongest host defense response to JEV infection and warrants further research to confirm its effects on JEV infection.

### 2.4. Validation of Deep Sequencing Results by RT-qPCR

**Figure 4:**
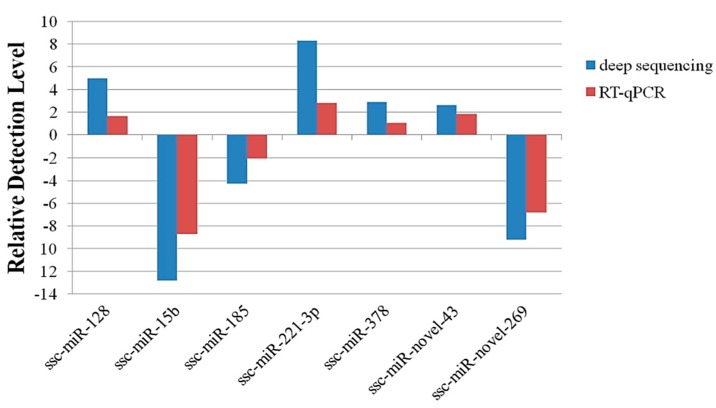
Validation of miRNA expression by RT-qPCR.

RT-qPCR was used to confirm the sequencing data. Seven candidate miRNAs were randomly selected: two novel miRNAs (ssc-miR-novel-43 and ssc-miR-novel-269) and five known miRNAs (ssc-miR-128, ssc-miR-15b, ssc-miR-185, ssc-miR-221-3p and ssc-mir-378). The miRNA primers are shown in Table 2. The expression levels of the miRNAs determined with deep sequencing and RT-qPCR were compared (Figure 4). On the whole, the relative expression levels of the selected miRNAs detected with RT-qPCR were lower than the relative expression levels determined with deep sequencing. The difference may explain the difference in the sensitivity of the two methods.

**Table 2:** Primers used to amplify miRNAs with RT-qPCR.

Primer	Sequence (5'-3')
ssc-miR-128-forward	TCACAGTGAACCGGTCTCTTT
ssc-miR-15b-forward	TAGCAGCACATCATGGTTTACA
ssc-miR-185-forward	TGGAGAGAAAGGCAGTTCCTGA
ssc-miR-221-3p-forward	AGCTACATTGTCTGCTGGGTTT
ssc-miR-378-forward	ACTGGACTTGGAGTCAGAAGGC
ssc-miR-novel-43-forward	TTCAAGTAACCCAGGATAGGCT
ssc-miR-novel-269-forward	TACCCATTGCATATCGGAGTTG
miR-reverse	GTCGGTGTCGTGGAGTCG
U6-forward	TCGCTTTGGCAGCACCTAT
U6-reverse	AATATGGAACGCTTCGCAAA
Poly(T) adapter	GTCGGTGTCGTGGAGTCGTTTGCAATTGCACTGGATTTTTTTTTTTTTTTTTTV

V = A, G, C.

### 2.5. GO Analysis of Predicted Targets of Differentially-Expressed miRNAs 

**Figure 5:**
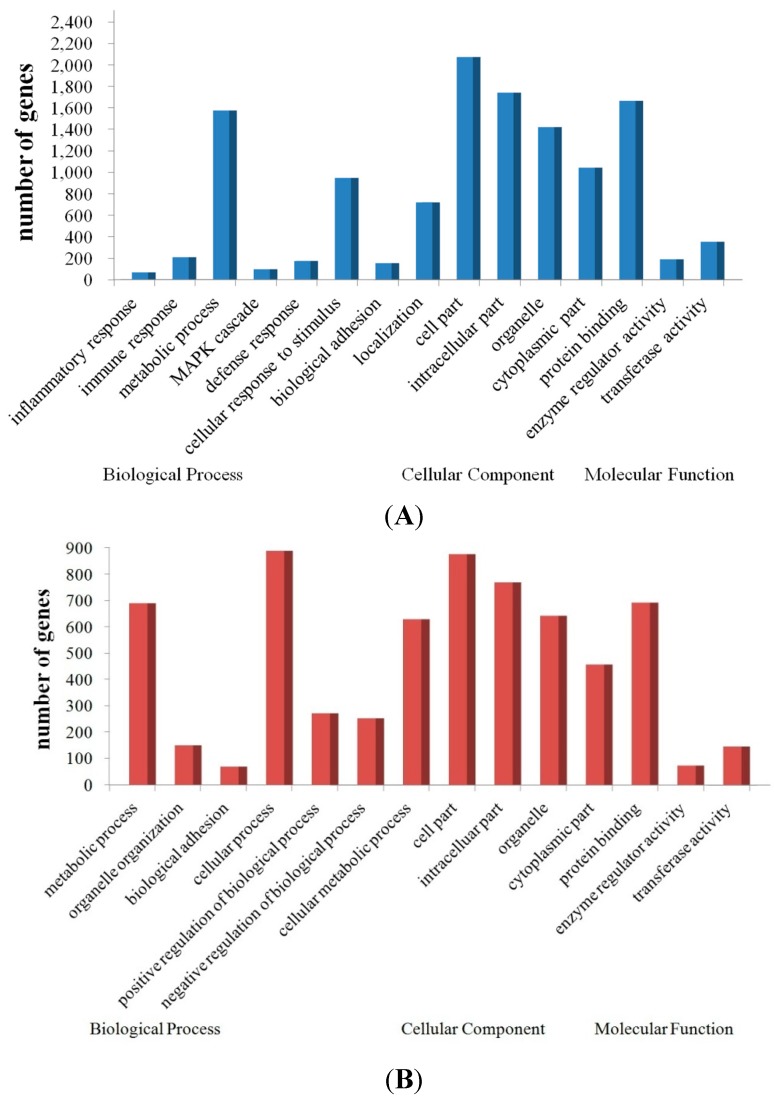
GO annotation of the predicted miRNA target genes. The figure shows the GO annotation of the upregulated genes (**A**) and downregulated genes (**B**) in biological processes, cellular components and molecular functions.

To further our understanding of the physiological functions of the dysregulated miRNAs in response to JEV infection, the potential targets of the up- and down-regulated miRNAs were predicted with two commonly-used miRNA target prediction software programs: TargetScan and miRanda. We found tens of thousands of putative targets in the combined outputs of both programs. All of the predicted target genes of the up- and down-regulated miRNAs were then subjected separately to GO analysis. More upregulated genes (targets of the downregulated miRNAs) than downregulated genes (targets of the upregulated miRNAs) were successfully annotated with the GO analysis. The GO category analysis revealed that the upregulated genes fell into the following categories: inflammatory response, immune response, defense response and stimulus response. The main GO categories for the downregulated genes were cellular process, metabolic process and regulation of biological process (Figure 5). Activation of these biological processes (especially related to inflammatory and immune response) suggests a strong antiviral response of the host, but may also contribute to JEV pathogenesis, resulting in encephalitis.

miRNAs have been shown to play important roles in the regulation of immune response, including the differentiation, proliferation, cell fate determination, function of immune cells and inflammatory mediator release, as well as the intracellular signaling pathways [44,45]. A previous study has shown that JEV has evolved a variety of strategies to evade the host’s immune responses, with a major focus on the inhibition of interferon, complement, natural killer (NK) cell, B-cell, and T-cell responses [46]. Therefore, it is important to identify candidate miRNAs associated with the immune response of host in JEV infection. Since the discovery of the role of miRNAs in the immune response, increasing numbers of immune-related miRNAs have been identified and profiled. These include miR-10, miR-101, miR-106a, miR-126, miR-142-3p, miR-146, miR-150, miR-155, miR-17-92 cluster (seven members: miR-17-5p, -17-3p, -18a, -19a, -20a, -19b and -92a), miR-181a, miR-196b, miR-21, miR-221, miR-223, miR-326, miR-34 ,miR-424, miR-9, miR-98, the let-7 family (nine members: let-7, mir-48, -84, -241, -265, -793, -794, -795 and -1821), and so on [47,48,49]. In this study, we identified a subset of miRNAs (including miR-10a-5p, miR-10a-3p, miR-10b, miR-101, miR-126-5p, miR-142-5p, miR-155-5p, miR-17-3p, miR-18a, miR-19a, miR-181a, miR-196b, miR-221-3p, miR-98, let-7d-3p, let-7d-5p) that are significantly differentially expressed after challenge with JEV. Particularly, It has been found that miR-10b has immune evasive functions [50]. These results suggest that JEV may utilize these immune-related miRNAs, contributing to immune evasion, resulting in persistent infection.

In addition to the miRNAs directly affecting immune responses, other miRNAs can indirectly mediate the host’s immune responses. For example, autophagy can reduce the innate antiviral immune response, resulting in the evasion of the host’s immune responses, and promote cell survival during JEV infection [51]. Studies have shown that a number of miRNAs relate to autophagy [52]. In this study, the miRNAs associated with autophagy, including miR-30a/c, miR-374a, miR-195, miR-101, miR-181a, miR-98, miR-142, miR-196, miR-210, miR-17 and miR-155, were identified. It was indicated that these miRNAs may function in JEV infection by regulating the autophagy pathway, and JEV replication may be indirectly regulated by these miRNAs.

## 3. Experimental Section

### 3.1. Cells and Virus

PK-15 cells were purchased from the China Center for Type Culture Collection (Wuhan, China) and cultured in RPMI 1640 supplemented with l-glutamine, penicillin (100 U/mL), streptomycin (100 mg/mL), fungizone (4 mg/mL) and 10% fetal bovine serum (FBS) at 37 °C under 5% CO_2_. The JEV-SC strain, stored at the Key Laboratory of Animal Diseases and Human Health of Sichuan Province, Ya’an, China, was used in this study. Monolayer cells were washed three times with phosphate-buffered saline (PBS), then infected with JEV at a multiplicity of infection (MOI) of 1 and incubated at 37 °C for 1 h with rocking every 15 min. After 1 h, 5 mL of fresh medium containing 2% FBS were added to the infected cells. Cells not infected with JEV were used as the control. Each group was performed in triplicates. The JEV-infected and -uninfected cells were harvested at 24 h post infection (hpi) and pooled separately for subsequent total RNA extraction.

### 3.2. RNA Isolation and Illumina Sequencing

The total RNA was extracted from each group of cells using TRIzol reagent (Life Technologies, Gaithersburg, MD, USA), according to the manufacturer’s instructions. The RNA concentration and purity were determined by measuring the absorbance at 260 nm (A_260_) and the A_260/A280_ ratio, respectively, using a NanoDrop ND-1000 spectrophotometer (Thermo Scientific, Wilmington, DE, USA). The two RNA samples were then sent to Kangcheng Bio-tech Inc. (Shanghai, China) for Illumina sequencing of the small RNAs, as follows. Adapters were first ligated to the 5' and 3' ends of the total RNAs, and the ligated samples were used as the templates for cDNA synthesis. The cDNA was then amplified by PCR to enrich the libraries. Following enrichment, the PCR products (~120–140 bp) were purified from a PAGE gel and their quality and concentrations confirmed with an Agilent 2100 Bioanalyzer (Agilent Technologies, Palo Alto, CA, USA). The DNA fragments in the libraries were then used for cluster generation and sequencing on an Illumina HiSeq 2000 instrument (Illumia Inc., San Diego, CA, USA).

### 3.3. Analysis of Sequencing Data

After sequencing, the images generated by the sequencer were analyzed, and base calling was performed with the Off-Line Basecaller software (OLB V1.8.0, Illumia Inc., San Diego, CA, USA) to produce digital-quality data. The small RNA sequencing reads obtained were then subjected to the following processes: (1) reads were filtered, and the low-quality reads were removed with the Solexa Chastity quality filter; (2) the adapter sequences were trimmed; (3) only trimmed reads longer than 15 nt were retained; and (4) low two-copy sequences were discarded. The filtered short reads were then mapped to the Rfam database (available online: http://rfam.janelia.org/), Repbase database (available online: http://www.girinst.org/repbase/) and NCBI database (available online: http://www.ncbi.nlm.nih.gov/). After the small RNAs filtered with the abovementioned processes were excluded, the remaining short reads were mapped to the known miRNA precursors, and the mature miRNAs were deposited in miRBase 19.0 (available online: http://www.mirbase.org/). The unmappable sequences not mapped to miRBase 19.0 were used to predict potentially novel candidate miRNAs with the Mfold RNA folding prediction web server (available online: http://mfold.rna.albany.edu/).

### 3.4. Identification of Differentially-Expressed miRNAs

To compare the differential miRNA expression in JEV-infected and -uninfected PK-15 cells, the numbers of raw tags in each library were normalized to tags per million of the total miRNA reads. To exclude from the analysis those miRNAs expressed at very low levels, we added a given low number (10 units) to each miRNA value. Differentially-expressed miRNAs were determined according to the criterion of a fold change of ≥2 in the sequence counts between libraries.

### 3.5. Reverse Transcription-Quantitative Real-Time PCR (RT-qPCR)

RT-qPCR was used to confirm the expression of the miRNAs identified with deep sequencing. The RNA samples used for the RT-qPCR assays were the same as those used for the deep sequencing experiments. Total RNA was first polyadenylated with polyA polymerase and then reverse transcribed to cDNA with a poly(T) adapter primer, using the GeneAmp PCR System 9700 (Applied Biosystems, Foster City, CA, USA). Selected miRNAs were subjected to RT-qPCR using the ViiA 7 Real-time PCR System (Applied Biosystems). The PCR reaction mixture (10 μL) consisted of 2 μL of cDNA, 5 μL of 2× SYBR Green PCR master mix, 0.5 μL each of the microRNA-specific forward primer, a universal reverse primer (10 μM) and 2 μL of nuclease-free water. The cycling parameters were 95 °C for 10 min followed by 40 cycles of 95 °C for 15 s and 60 °C for 60 s. U6 was used as the endogenous control for normalization. Three independent biological replicates were performed. The relative expression levels were calculated with the 2^−ΔΔ*C*t^ method.

### 3.6. miRNA Target Prediction

The potential target genes of the differentially-expressed miRNAs were predicted with two miRNA target prediction algorithms, miRanda (available online: http://microorna.sanger.ac.uk/sequencs/) and TargetScan (available online: http://www.targetscan.org/). The list of potential target genes was filtered with the following higher stringency criteria: (1) a match between nucleotides 2–8 of the miRNA and the target sequence or (2) a match between nucleotides 2–7 and 13–16 of the miRNA and the target sequence (G:U wobble tolerance); (3) high local AU content near the site; and (4) sited away from the centers of long UTRs.

### 3.7. Gene Ontology Analysis

The Gene Ontology program (available online: http://www.geneontology.org) was used for GO annotation and enrichment analysis of the miRNA target genes based on three ontologies: cell component, biological process and molecular function. The false discovery rate (FDR) based on the Benjamini–Hochberg method was evaluated using the default parameters to adjust the *p*-values. Genes with FDR ≤0.05 were considered to be significantly enriched in the target candidate genes.

## 4. Conclusions

In summary, using deep sequencing, we have described for the first time a set of miRNAs dysregulated in PK-15 cells by JEV infection. The differentially-expressed miRNAs may induce a strong antiviral response in the host, but may also contribute to the pathogenesis of JEV. Although the exact roles of these dysregulated miRNAs during JEV infection are still unknown, our findings describe the JEV virus-host interaction at the miRNA level. In future studies, we will investigate the functions of these dysregulated miRNAs by correlating the miRNA and mRNA profiles in the same pathogen-infected samples and will clarify the mechanisms underlying the mediation by these candidate miRNAs of the host-virus interactions during JEV infection. Because miRNA expression depends on both tissue type and development stage, much work will be required for a comprehensive understanding of the processes underlying the development of the encephalitis caused by JEV infection in different hosts.

## Supplementary Materials

Supplementary materials can be found at http://www.mdpi.com/1422-0067/16/01/2204/s1.
